# Water-Borne Cues of a Non-Indigenous Seaweed Mediate Grazer-Deterrent Responses in Native Seaweeds, but Not Vice Versa

**DOI:** 10.1371/journal.pone.0038804

**Published:** 2012-06-12

**Authors:** Hee Young Yun, Aschwin H. Engelen, Rui O. Santos, Markus Molis

**Affiliations:** 1 Section Functional Ecology, Biologische Anstalt Helgoland, Alfred-Wegener-Institute for Polar and Marine Research, Helgoland, Germany; 2 Marine Plant Ecology Research Group, Centre of Marine Sciences, University of Algarve, Faro, Portugal; Centro de Investigación y de Estudios, Mexico

## Abstract

Plants optimise their resistance to herbivores by regulating deterrent responses on demand. Induction of anti-herbivory defences can occur directly in grazed plants or from emission of risk cues to the environment, which modifies interactions of adjacent plants with, for instance, their consumers**.** This study confirmed the induction of anti-herbivory responses by water-borne risk cues between adjoining con-specific seaweeds and firstly examined whether plant-plant signalling also exists among adjacent hetero-specific seaweeds. Furthermore, differential abilities and geographic variation in plant-plant signalling by a non-indigenous seaweed as well as native seaweeds were assessed. Twelve-day induction experiments using the non-indigenous seaweed *Sargassum muticum* were conducted in the laboratory in Portugal and Germany with one local con-familiar (Portugal: *Cystoseira humilis*, Germany: *Halidrys siliquosa*) and hetero-familiar native species (Portugal: *Fucus spiralis*, Germany: *F. vesiculosus*). All seaweeds were grazed by a local isopod species (Portugal: *Stenosoma nadejda*, Germany: *Idotea baltica*) and were positioned upstream of con- and hetero-specific seaweeds. Grazing-induced modification in seaweed traits were tested in three-day feeding assays between cue-exposed and cue-free ( = control) pieces of both fresh and reconstituted seaweeds. Both *Fucus* species reduced their palatability when positioned downstream of isopod-grazed con-specifics. Yet, the palatability of non-indigenous *S. muticum* remained constant in the presence of upstream grazed con-specifics and native hetero-specifics. In contrast, both con-familiar (but neither hetero-familiar) native species reduced palatability when located downstream of grazed *S. muticum*. Similar patterns of grazer-deterrent responses to water-borne cues were observed on both European shores, and were almost identical between assays using fresh and reconstituted seaweeds. Hence, seaweeds may use plant-plant signalling to optimise chemical resistance to consumers, though this ability appeared to be species-specific. Furthermore, this study suggests that native species may benefit more than a non-indigenous species from water-borne cue mediated reduction in consumption as only natives responded to signals emitted by hetero-specifics.

## Introduction

Herbivory is an important factor in structuring ecological communities that is presumed to have a stronger effect in marine than in terrestrial habitats [1], [2]. Extreme grazing events may completely defoliate plants [3], or denude entire seascapes [4]. However, plants usually persist with variable success in the presence of grazers. Hence, rather than being simple and passive participants in their interaction with hungry consumers, plants can actively deter herbivores [5], [6], [7]. Understanding drivers and processes influencing the outcome of plant-grazer interactions is therefore a pivotal goal in ecology to improve predictions regarding community structure under current and future environmental conditions.

Plants may deploy anti-herbivory defences constitutively, as in the use of grazer repulsive secondary metabolites [3], [8]. According to theory, the efficacy of inducible defences is dependent on the predictability of future risk and the speed at which defensive traits are produced [8], [9]. Since grazing by smaller-sized herbivores like insects or gastropods is usually not lethal for larger vascular plants and seaweeds, expression of grazer-deterrent resistance within ecologically meaningful times is possible [10], [11]. Furthermore, tailoring resistance to actual threats may imply a selective advantage in plants if grazer-deterrent responses incur a metabolic cost [12]. Not surprisingly, inducible anti-herbivory defences are widespread in plants [10] and seaweeds [13] and can indirectly have great effects on herbivore species richness [7], inter-specific competition among herbivores [14], [15], and community structure [16]. Moreover, grazing by small (<2.5 cm) herbivores, known as meso-herbivores [17], has been shown to elicit emission of air- and/or water-borne risk cues in vascular plants and seaweeds [18], [19]. In addition, some seaweed species when damaged release gaseous volatile substances such as dimethylsulphide (DMS) or trimethylamine (TMA) that may function as grazer deterrents in the aqueous phase, as found for *Dictyota dichotoma* [20]. Other soluble organic compounds from seaweeds, such as alginate derivates, may also mediate species interactions [21]. The use of risk compounds probably evolved initially to optimise within-plant signalling to counter grazing [19]. Furthermore, plants and seaweeds with a complex anatomy and/or limited vascular system should gain a selective advantage from using risk signals, as these may prime undamaged parts allowing more rapid and systemic responses, e.g. to attacking grazers, than information transfer by internal transport systems [22]. The emission of risk cues to the environment would principally allow intra-specific signalling between adjoining con-specifics: it was shown, e.g. that undamaged corn (*Zea mays*) exposed to caterpillar (*Spodoptora exigua*) induced emission of volatile signals from adjacent grazed corn accelerated and intensified the expression of anti-herbivory traits, and therefore shortened the period of vulnerability once attacked [23]. Furthermore, optimising plant resistance by using emitted signals may allow attacked plants to save their defence budget [22]. Emitted signals, however, become public information that may be used in plant-plant communication, i.e. signals are supposedly positive for both emitter and receiver, or for eavesdropping, i.e. signals benefit the receiver but have neutral or negative effects on the emitting species [19], smoothing the way for mutualistic [24] and antagonistic indirect interactions [25].

Knowledge of antagonistic indirect effects mediated by risk cues is scarce for trophic interactions among plants and herbivores [19], and as far as we know, examples involving seaweeds are lacking. Hence, commonness of and patterns in plant-plant signalling in general and their existence in seaweeds in particular have yet to be determined [22]. The effective spatial range of centimetres reported for emitted risk compounds between terrestrial plants [25], for instance, suggests that plant-plant signalling should occur among specimens dwelling in close proximity. Consequently, plant-plant communication and eavesdropping should be more likely in species that establish dense mono-specific and mixed stands, respectively, than in species with isolated, remotely growing individuals. The ability to respond to risk cues may additionally be affected by the period of shared history. Ancient sympatry has been reported to result in more appropriate host counter-adaptations than is the case with recent sympatry [26]. Thus, the duration of sympatry should be particularly important in driving interactions between native and non-indigenous species. Knowledge of predator-prey interactions suggests that the ability of native gastropods responding to risk cues of a non-indigenous predatory crab manifested in a range of months to decades [27], [28]. Hence, establishment of the densely growing, non-indigenous brown seaweed *Sargassum muticum* along European shores since the 1980s may constitute a sufficiently long period of shared history to render plant-plant signalling possible for both native seaweeds and *S. muticum*. To explore this assumption, bio-assayed induction experiments were conducted in which *S. muticum* from two European shores and two respective local native seaweed species were exposed to grazing by a local isopod species. These experiments tested whether induced chemical resistance to herbivory was mediated by water-borne cues (i) between con-specific native seaweeds as well as *S. muticum* (intra-specific signalling), (ii) between *S. muticum* and native seaweeds (inter-specific signalling), and (iii) whether such responses show geographic variation.

## Results

### Intra-specific Signalling

#### Southern site

**Figure 1:**
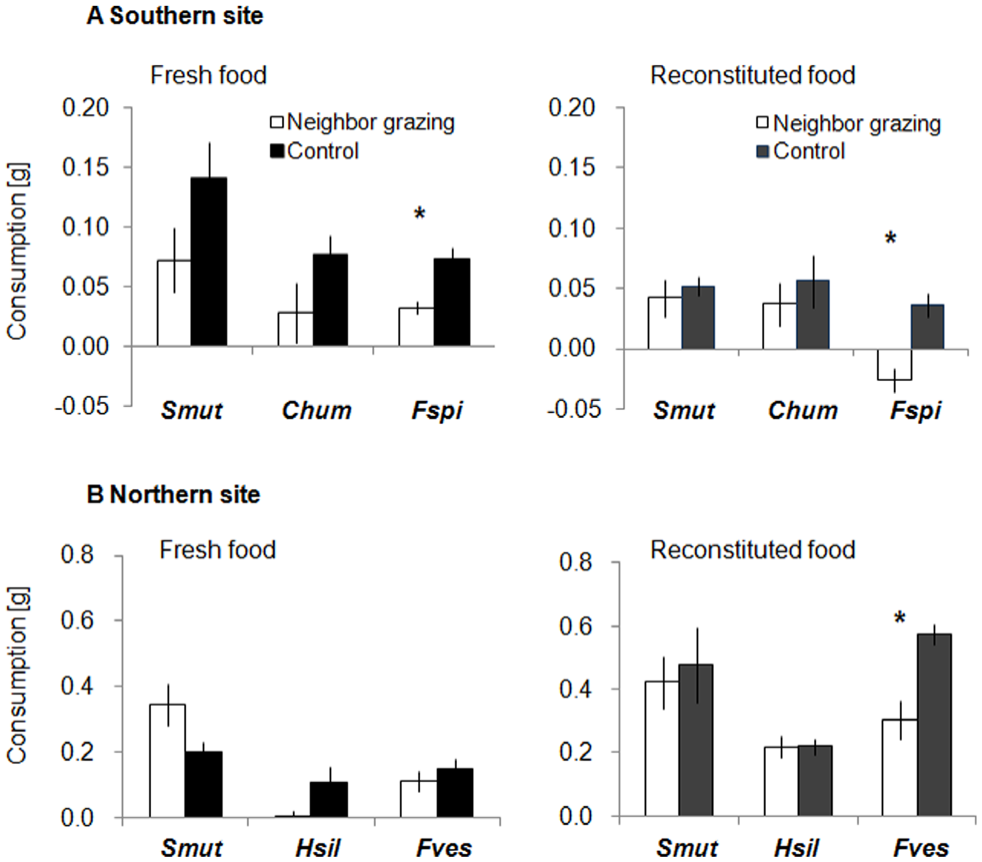
Effects of waterborne cues on conspecific seaweeds. Mean consumption (±SE) of fresh and reconstituted seaweed pieces by the isopod *Stenosoma nadejda* from the southern site (A) and *Idotea baltica* from the northern site (B) in two-choice feeding assays. The seaweed species *Sargassum muticum* (Smut), *Cystoseira humilis* (Chum), *Halidrys siliquosa* (Hsil), *Fucus spiralis* (Fspi) and *F. vesiculosus* (Fves) used in the assays were positioned downstream of grazed ( = cue-exposed) and ungrazed ( = control) con-specifics in the induction phase. Negative values in consumption of reconstituted food indicate lower losses in mass by consumption than increases in mass by non-consumptive effects, e.g. absorption of water. Almost identical consistency and same volume of reconstituted food will result in same magnitude of non-consumptive effects on wet mass change during assays and allow interpretation of preferences between treated and control pieces. Asterisks show significant differences in consumption between cue-exposed and control feed (n = 8). Note different scaling of ordinate between southern and northern sites.

Consumption by the isopod *Stenosoma nadejda* was not significantly different between pieces of *S. muticum* positioned downstream of grazed and ungrazed con-specifics in both bioassays using fresh and reconstituted *S. muticum* (fresh t_7_ = 1.40, p = 0.205; reconstituted t_7_ = 0.47, p = 0.653, Fig. 1A). Similarly, the palatability of fresh and reconstituted pieces of *Cystoseira humilis* located downstream of grazed con-specifics was not significantly different from the palatability of pieces located downstream of ungrazed *C. humilis* (fresh t_7_ = 1.41, p = 0.203; reconstituted t_7_ = 0.57, p = 0.589, Fig. 1A). In contrast, pieces of fresh *Fucus spiralis* located downstream of grazed con-specifics were on average 52% less palatable than pieces positioned downstream of ungrazed con-specifics (t_7_ = 2.98, p = 0.021, Fig. 1A). This pattern was also apparent in assays using reconstituted pieces of *F. spiralis* (t_7_ = 3.46, p = 0.011, Fig. 1A).

#### Northern site

The isopod *Idotea baltica* consumed fresh as well as reconstituted pieces of *S. muticum* located downstream of grazed and ungrazed con-specifics in equivalent amounts (fresh t_7_ = −2.18, p = 0.066; reconstituted t_7_ = 0.61, p = 0.563, Fig. 1B). Likewise, *I. baltica* showed no significant preference for either fresh or reconstituted pieces made of *Halidrys siliquosa* that were located downstream of ungrazed con-specifics over *H. siliquosa* pieces located downstream of grazed con-specifics (fresh t_7_ = 1.96, p = 0.091; reconstituted t_7_ = 0.30, p = 0.977, Fig. 1B). Fresh pieces made of *F. vesiculosus* positioned downstream of ungrazed con-specifics were also not significantly more palatable to *I. baltica* than pieces located downstream of grazed con-specifics (t_7_ = 1.00, p = 0.349, Fig. 1B). There was, however, a significant preference for ungrazed *F. vesiculosus* by 47% in corresponding reconstituted food assays (t_7_ = 3.54, p = 0.009, Fig. 1B).

### Inter-specific Signalling

#### Southern site

**Figure 2:**
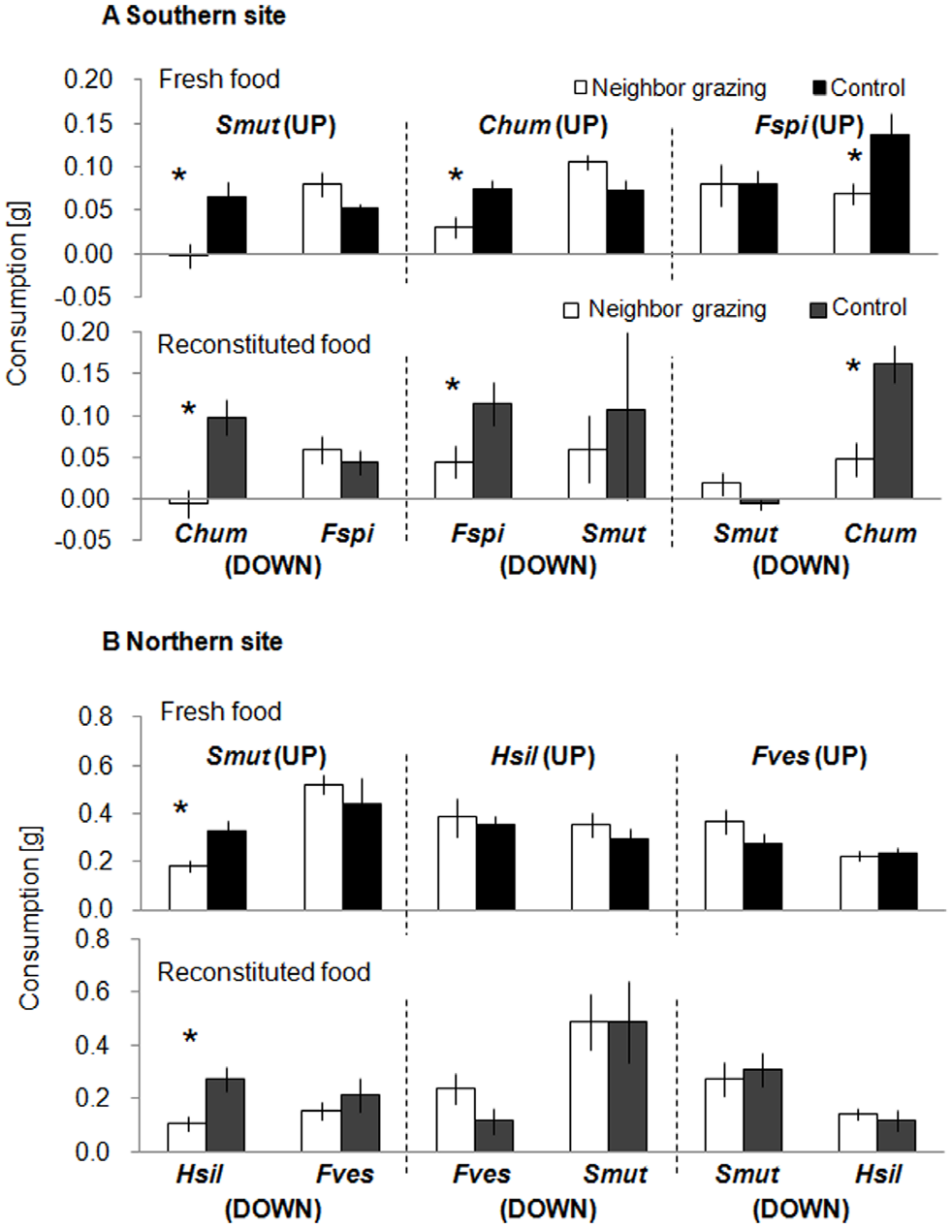
Effects of waterborne cues on heterospecific seaweeds. Mean consumption (±SE) of fresh and reconstituted seaweed pieces by the isopod *Stenosoma nadejda* from the southern site (A) and *Idotea baltica* from the northern site (B) in two-choice feeding assays. The seaweed pieces used in the assays were positioned downstream of grazed ( = cue-exposed) and ungrazed ( = control) hetero-specifics in the induction phase. ‘Up’ and ‘down’ indicate the position of the seaweed species in aquaria during the induction phase. Note different scaling of ordinate between southern and northern sites (n = 8). Interpretation of symbols, abbreviations, and explanation of negative values in assays using reconstituted food as in Fig. 1.

S. *nadejda* showed no significant preference, either in bioassays with fresh or with reconstituted *S. muticum*, between *S. muticum* pieces located in the induction phase downstream of grazed and ungrazed *F. spiralis* (fresh t_7_ = 0.06, p = 0.958; reconstituted t_7_ = 1.77, p = 0.121, Fig. 2A). Likewise, the consumption of *S. muticum* kept downstream of grazed *C. humilis* pieces was not significantly different from that of *S. muticum* pieces located downstream of ungrazed *C. humilis* (fresh t_7_ = −1.73, p = 0.127; reconstituted t_7_ = 0.47, p = 0.656, Fig. 2A). In contrast, fresh and reconstituted pieces of *C. humilis* were both significantly more palatable in bioassays when positioned downstream of ungrazed *S. muticum* in the induction phase than when positioned downstream of grazed *S. muticum* (fresh t_7_ = 3.40, p = 0.011; reconstituted t_7_ = 2.99, p = 0.020, Fig. 2A). Similarly, fresh *C. humilis* pieces that were positioned downstream of ungrazed *F. spiralis* in the induction phase were consumed in bioassays significantly, i.e. 2 times, more on average than *C. humilis* pieces located downstream of grazed *F. spiralis* (t_7_ = 2.56, p = 0.037, Fig. 2A). This effect was considerably more pronounced in assays using reconstituted *C. humilis* pieces (t_7_ = 3.65, p = 0.008, Fig. 2A). Isopods significantly preferred fresh pieces and reconstituted food made of *F. spiralis* pieces that were positioned downstream of ungrazed *C. humilis* pieces by 2.4- and 2.5-fold, respectively, on average compared to *F. spiralis* that was positioned downstream of grazed *C. humilis* (fresh t_7_ = 2.73, p = 0.029; reconstituted t_7_ = 3.14, p = 0.016, Fig. 2A). Yet, isopods showed no preference between pieces of *F. spiralis* that were located downstream of grazed and ungrazed pieces of *S. muticum* (fresh t_7_ = −1.82, p = 0.112; reconstituted t_7_ = −0.55, p = 0.602, Fig. 2A).

#### Northern site

Isopods consumed *H. siliquosa* pieces that were positioned downstream of ungrazed *S. muticum* significantly, i.e. 2.5 (fresh pieces) and 1.8 times (reconstituted food), more than those located downstream of grazed *S. muticum* (fresh t_7_ = 2.92, p = 0.023; reconstituted t_7_ = 3.13, p = 0.017, Fig. 2B). Yet, in all other assays isopods showed no significant feeding preferences (all t_7_<0.91, p>0.157, Fig. 2B).

## Discussion

Both *Fucus* species reduced their palatability when nearby con-specifics were grazed by isopods. In contrast, the palatability of the non-indigenous brown seaweed *S. muticum* did not change either when located downstream of grazed con-specific or of native hetero-specific brown seaweeds. Furthermore, both con-familiar, but neither hetero-familiar native seaweed species reduced palatability when located downstream of grazed *S. muticum*. These patterns were observed at both European shores and were almost identical between assays using fresh and reconstituted seaweeds. Although the nature of the water-borne risk cues mediating trophic interactions between grazers and seaweeds is still unknown, several other studies working on seaweed-seaweed interactions assumed water-borne cues to evolve as signals in aquatic environments [18], [29], [30].

### Intra-specific Signalling

The higher palatability of reconstituted food made of *F. vesiculosus* positioned downstream of ungrazed than of *I. baltica-*grazed con-specifics suggests an induction of chemical rather than structural anti-herbivory defences that was mediated by water-borne cues. This finding corroborates Rohde et al. [31], who additionally confirmed, like others, but in contrast to our study, this ability of *F. vesiculosus* with assays using fresh specimens [29]. We think that this mismatch between results of fresh and reconstituted assays, which was the only case in the entire study, may not represent an artefact from processing reconstituted food as this should have resulted in a higher frequency of mismatches. A single mismatch out of a total number of 18 assays is, however, likely due to chance alone (18×0.05 = 0.9). Moreover, this study documents for the first time that water-borne cues emitted from nearby grazed con-specifics may induce anti-herbivory defences in *F. spiralis*. At present, experimental evidence on plant-plant signalling is scarce for both vascular plants [19] and seaweeds. There are, for instance, <10 examples from seaweeds, all including species of the families Fucaceae (*Ascophyllum nodosum*: [18]; *F. vesiculosus* and *F. spiralis*: e.g. both this study) and Dictyotacea [30]. Theory suggests [9] and empirical data [23] support an advantage for species with an ability to use plant-plant signalling since risk cues permit early detection of and priming against future grazer attacks. This may intensify and/or accelerate induction of anti-herbivory defences and hence shorten the period of vulnerability [19], [22].

Grazing by isopods reduced palatability in nearby ungrazed con-specifics in only one of the three seaweed species tested at each site. This suggests that intra-specific signalling by water-borne cues may depend on seaweed species. This species specificity may probably be due to a distance-dependent efficacy of risk cues. Defoliated alder (*Alnus glutinosa*), for instance, affected resistance expression only in con-specifics growing at a distance of a few metres [32]. This suggests that the function of risk cues may be dose-dependent [19]. Consequently, plant species with an aggregated distribution of specimens should benefit more from risk cue signalling than species with a scattered distribution of isolated specimens. Feeding preferences of isopods from both study sites confirmed this assumed pattern for most species tested in this study. On the one hand, both cue-sensitive species occur at the study sites either in dense stands (*F. spiralis*) or show a clumped distribution (*F. vesiculosus*) in which neighbours thrive in immediate proximity. On the other hand, responses to water-borne cues emitted from nearby grazed con-specifics were lacking in *S. muticum* and *C. humilis*, despite their occurrence in dense stands at the study sites. At least two explanations for this observed cue-insensitivity seem possible. First, both species probably counter grazing losses by compensatory growth. Growth rates of up to 46 and 80 cm per month in spring, i.e. at times when grazer density increases, have been reported for *S. muticum* at the southern (unpubli. data AE Engelen) and northern study site [33], respectively. Moreover, growth rates are similar in both seaweed species, along with palatability to the isopod *S. nadeja* [34]. Second, both species may use constitutive defences, although fast growing plants are predicted to invest less in constitutive defences than slower-growing plants [22]. Moreover, in this study *S. muticum* was consumed at least as much as undefended seaweeds, i.e. controls of *Fucus* species. Finally, adjacent specimens of the cue-insensitive species at the north European site, *H. siliquosa*, are separated by >3 m and persist in areas where *S. muticum* forms dense stands [33]. The minimum distance between adjacent isolated con-specific individuals was larger than the reported range at which risk cues are functional around emitting individuals, e.g. 60 cm in sagebrush [35]. Future tests on spatial limitations and dose dependency in the efficacy of water-borne cues are needed to clarify distance-dependence of risk cues.

### Inter-specific Signalling

The palatability of fresh and reconstituted pieces of *C. humilis* was lower downstream of grazed than ungrazed *S. muticum*. This suggests an induction of chemical anti-herbivory defences in a hetero-specific seaweed in response to water-borne cues emitted by *S. muticum*. However, because the palatability of *S. muticum* did not change significantly when located downstream of either grazed con-specifics or grazed *C. humilis*, there was no reciprocal benefit for *S. muticum* at the southern European site. Similarly, the palatability of *H. siliquosa* at the northern European site was reduced when located downstream of *S. muticum*, with no reciprocal beneficial effects. These results suggest specificity of inducible effects, which has been already documented, for instance, in the induction of anti-herbivory defences of directly grazed vascular plants and seaweeds [15], [36].

Responses by hetero-specific native seaweeds to signals emitted by non-indigenous *S. muticum* represent to our knowledge the first report on inter-specific signalling in seaweeds. Due to the experimental set-up, i.e. uni-directional flow of seawater, downstream located cue-receiving seaweeds were able to perceive signals from upstream located cue-emitting seaweeds but not *vice versa*. Thus, results suggest that *S. muticum* was eavesdropped by native seaweeds under experimental conditions, but this may be inapplicable under natural conditions. The eavesdropping aspect is, however, supported by missing reciprocal effects in this study, i.e. no change in *S. muticum* palatability when it was located downstream of native species. Experimental evidence on eavesdropping also exists for terrestrial plants [37], [38]. Eavesdropping should be especially advantageous for isolated specimens that are surrounded by dense emitter stands associated with generalist consumers. Both isopod species used in this study can be characterised as generalists on the respective shores since they displayed similar consumption rates between *S. muticum* and native seaweed species, including *F. spiralis*, *C. humilis*, and *F. vesiculosus* [34], [39]. Hence, consumption of *S. muticum* by isopods should provide a reliable indication of the future risk of grazing by isopods for the adjacently growing native seaweeds used in this study and vice versa. However, this study offers no evidence that *S. muticum* was able to respond to inter-specific signals of any of the tested native seaweed species. The short period for co-evolution does not seem to explain cue immunity by *S. muticum* as some native species gained the ability to respond to signals emitted by *S. muticum* within the same period. It is more likely that *S. muticum* principally refrains from using water-borne cues to induce defences against its consumers. This explanation is supported by the unchanged palatability of *S. muticum* in the intra-specific signalling experiment and the possibility that this fast-growing species compensates grazing losses with growth (see above discussion).

At both sites, only non-*Fucus* native seaweed species responded to signals emitted by isopod-grazed *S. muticum*. The specificity of inter-specific signalling between *S. muticum* and neighbouring, native seaweeds corroborates results by another study [40], which tested several forbs growing near sagebrush. The fact that both *Fucus* species lowered their palatability when positioned downstream of grazed con-specifics clearly indicates that lack of plant-plant signalling between *S. muticum* and *Fucus* species was not due to general insusceptibility to water-borne cues of the latter. Differences in cue concentration between the intra-specific and inter-specific signalling experiment seem unlikely to explain this differential ability of native seaweed species to respond to *S. muticum* signals, as consumption rates of upstream located *C. humilis* as well as *H. siliquosa* were not significantly different between both experiments. Several other explanations, however, seem possible. First, patterns of plant-plant signalling may be explained by the spatial distances between seaweeds in the field. At both sites *S. muticum* and the cue-sensitive native species (*C. humilis* and *H. siliquosa*) exist in immediate proximity, while cue-insensitive natives grow at a distance of metres (*F. spiralis*, southern site) to tens of metres (*F. vesiculosus*, northern site) apart. Similarly, results from vascular plants indicate that the efficacy of risk cues was negatively correlated with distance between emitter and receiver [32]. Where specimens of one species dwell scattered within dense stands of a second species the former will probably encounter a reliable cue regime, at least of generalist consumers, as these will consume specimens of both seaweeds. This would explain why *H. siliquosa* in our study responded to signals emitted by *S. muticum* but not to potential cues from more distantly located conspecifics. Second, different exposure intensity to *S. muticum* cues might have occurred between native seaweeds. While cue-sensitive species cohabit with *S. muticum* in permanently submersed habitats, i.e. tide pools and the shallow subtidal, cue-immune species inhabit the intertidal. Consequently and in contrast to cue-sensitive species, both cue-immune species were disconnected from *S. muticum* signals during low tide. Assuming that cue efficacy is dose-dependent, contact-free periods might provoke invalidity of *S. muticum* cues for intertidal species as induction models suggest that evolution of inducible responses is dependent on cue reliability [9]. Finally, results from the northern but not from the southern European site allow speculating that relatedness and the ability to plant-plant signalling may correlate positively because water-borne cues only affected seaweed palatability in assays using con-specific and con-familiar species. At present it is unknown whether close relatives are more effective communicators than genetically less similar plants [22]. As the number of seaweed species included in our study was too small to draw resilient conclusions about this relationship, more comparisons are needed to clarify whether the ability of inter-specific signalling is kinship-dependent.

This study is to our knowledge the first documentation of inter-specific signalling in seaweeds. The unidirectional ability of plant-plant signalling between native species and *S. muticum*, through which only anti-herbivory responses of the former are tailored, provides an example where a non-indigenous species may benefit native species. Increasing, knowledge about plant-plant signalling may elucidate additional and effective drivers that structure communities, will lead to a more comprehensive understanding on, and improved prediction capacities of the ecology of communities for the present and in a changing world.

**Table 1:** Average (±SD) wet mass of apical pieces of seaweeds used in intra-specific signalling experiments at the southern (Portugal) and northern (Germany) European study sites.

Species name	Wet mass [g]	Study site
*Sargassum muticum*	1.181 (±0.159)	Portugal
*Sargassum muticum*	1.167 (±0.204)	Germany
*Cystoseira humilis*	0.993 (±0.112)	Portugal
*Halidrys siliquosa*	1.790 (±0.189)	Germany
*Fucus spiralis*	0.701 (±0.118)	Portugal
*Fucus vesiculosus*	1.693 (±0.216)	Germany

**Table 2:** Average (±SD) wet mass of apical pieces of seaweeds used in inter-specific signalling experiments at the southern (Portugal) and northern (Germany) European study sites.

Cue-donor species	Cue-recipient species	Wet mass [g]	Study site
*Sargassum muticum*	*Cystoseira humilis*	0.859 (±0.260)	Portugal
*Sargassum muticum*	*Fucus spiralis*	0.607 (±0.162)	Portugal
*Sargassum muticum*	*Halidrys siliquosa*	1.600 (±0.306)	Germany
*Sargassum muticum*	*Fucus vesiculosus*	1.190 (±0.232)	Germany
*Cystoseira humilis*	*Sargassum muticum*	0.825 (±0.282)	Portugal
*Cystoseira humilis*	*Fucus spiralis*	0.741 (±0.294)	Portugal
*Halidrys siliquosa*	*Sargassum muticum*	1.645 (±0.534)	Germany
*Halidrys siliquosa*	*Fucus vesiculosus*	1.991 (±0.418)	Germany
*Fucus spiralis*	*Sargassum muticum*	0.874 (±0.279)	Portugal
*Fucus spiralis*	*Cystoseira humilis*	0.921 (±0.254)	Portugal
*Fucus vesiculosus*	*Sargassum muticum*	0.679 (±0.175)	Germany
*Fucus vesiculosus*	*Halidrys siliquosa*	1.404 (±0.157)	Germany

## Materials and Methods

### Collection Sites and Organisms

Three seaweed and one herbivore species each were collected at two NE Atlantic sites located >2000 km apart: (i) Praia de Queimado, SW Portugal (37° 49' N, 8° 47' W, Southern Europe) and (ii) Nordwatt, Helgoland, Germany (54° 11' N, 7° 52' E, Northern Europe). At both sites the non-indigenous wireweed (*S. muticum*) was collected together with one con-familiar (Portugal: *C. humilis*, Germany: *H. siliquosa*) and one hetero-familiar (Portugal: *F. spiralis*, Germany: *F. vesiculosus*) seaweed species. The southern European site is located on a wave-sheltered shore with semidiurnal tides having a range of 3 m. Average (±SE) seawater temperature at this fully marine site was 16.9 (±0.5)°C during the study period. Dense stands of perennial brown seaweeds, including *S. muticum* were located inside and along rock pool edges. *S. muticum* was first observed at the southern European site in 2002 [41], where it co-occurs with *C. humilis* inside rock pools, while *F. spiralis* grows outside rock pools on emergent rock. The isopod *S. nadejda* was collected from rock pools, where this seaweed-associated mesograzer is abundant [42]. The northern European site, Nordwatt, is a semi-exposed intertidal rocky shore on Helgoland, Germany. The shore is characterised by large sandstone terraces and semidiurnal tides having an average range of 2.35 m. Seawater temperature was, on average, 18.30 (±1.99)°C during the experiments. Benthic assemblages were dominated by perennial brown seaweeds, such as *Fucus* species in the intertidal and *Laminaria* species in the subtidal. The pseudo-perennial *S. muticum* was first encountered on Helgoland in 1989 [43]. Around May *S. muticum* quickly develops dense stands in the sheltered shallow subtidal (<3 m), interspersed by the perennial sea oak (*H. siliquosa*). In contrast, the perennial bladder wrack (*F. vesiculosus*) inhabits the mid intertidal, i.e. up to tens of metres apart from subtidal habitats where the two other species dwell. The isopod *I. baltica* used as the grazer was collected around Helgoland from *Fucus* spp. and *S. muticum*.

**Figure 3:**
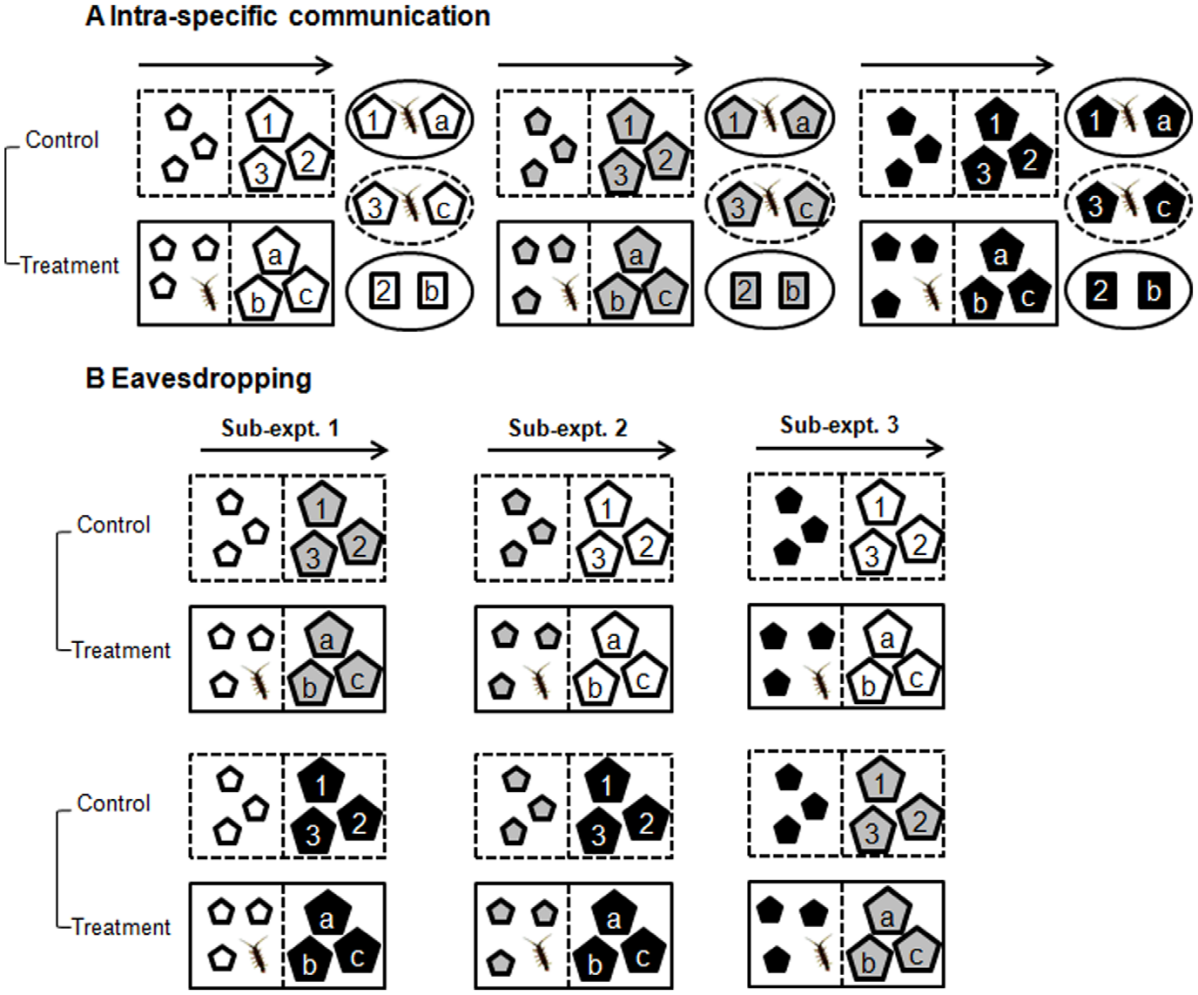
Schematic experimental set-up (displayed for one replicate). Induction of anti-herbivore defences in non-damaged seaweeds was tested in response to water-borne cues from nearby grazed con-specifics (A) and hetero-specifics (B). Aquaria (large rectangles) were supplied by a unidirectional flow of seawater (indicated by arrow) and were divided by a net (dashed line) into upstream and downstream compartments. Small and large pentagons designate seaweed pieces serving as donors and recipients of water-borne cues, respectively. Seaweed species are differentiated by the colours of the pentagons. After 4 d of acclimatisation, grazers were added to treatment aquaria (solid line) and absent in control aquaria (dotted line) during the subsequent 12 d induction phase. Numbers and letters indicate different use of downstream treatment and control seaweed pieces, respectively, in fresh ( = 1 or a) and reconstituted ( = 2 or b) feeding assays, and as autogenic controls ( = 3 or c). Circles with solid and stippled lines illustrate feeding arenas with and without grazers ( = autogenic controls), respectively, in which fresh or reconstituted (quadrates) pieces of seaweed were offered. For clarity, feeding arenas were only shown for intra-specific communication experiments.

### Experimental Design and Set-up

To assess the generality of induced seaweed responses by water-borne cues, experiments were run with organisms from both the southern and northern European site. At each site two experiments (n = 8) were conducted, each divided into three sequential phases: acclimation (4 d), induction (12 d), and bio-assay (3 d). The first experiment started on 7 April and 30 June 2007 at the southern and northern site, respectively, testing whether water-borne cues from grazed seaweeds induce anti-herbivory responses in con-specifics ( = intra-specific signalling). The second experiment assessed whether water-borne cues from grazed seaweeds induce anti-herbivory defences in hetero-specifics ( = inter-specific signalling). Due to limited laboratory space, the inter-specific signalling experiment was divided into three consecutive sub-experiments (57 d total experimental period), starting on 1 May and 2 July 2007 at the southern and northern site, respectively.

The day the experiments started, up to 400 isopods and 8 specimens of each seaweed species were collected and transported to the laboratory within 1 h. Due to the small size of *F. spiralis*, 24 to 36 specimens were needed to obtain the full number of seaweed pieces in experiments. In the laboratory macroscopic epibionts were gently removed from all seaweeds with a sponge to minimize confounding effects from e.g. ‘co-consumption’ or ‘protective coating’ [44]. Then all apical pieces (Table 1 & 2) needed for one replicate were cut from one specimen (except *F. spiralis*, where only 2 pieces were cut from each specimen) and individually marked with coloured threads. The pieces of each specimen (multiple *F. spiralis* individuals) were allocated to transparent plastic aquaria (Fig. 3, detailed description below), containing 2 L (southern site) or 8 L (northern site) of seawater. Each aquarium was divided with a plastic mesh (1 mm mesh size) into equally sized upstream and downstream compartments. The aquaria were individually supplied with a unidirectional flow of cotton-filtered seawater from the nearby sea at an average flow rate of 120 (southern site) or 300 ml min^−1^ (northern site). These differences in set-ups were due to technical constrains but did not seem to interfere with results obtained at each site, though we cannot rule out any undetected differences. Generated flow-rates represent, however, intermediate levels of dilution compared to other studies testing the effects of water-borne cues on seaweed palatability [18], [31], [45]. Fluorescent lamps (58 W Osram at southern site, 36 W Philips at northern site) irradiated aquaria in a 12 h:12 h light-dark cycle with, on average, 1210 lux (PeakTech light meter 5025) at the southern and 34.4±2.5 µmol^−2^ s^−1^ (LI-COR broadband sensor UWQ 8534) at the northern site. Irradiance in the laboratory simulated ambient PAR levels at 1 m water depth during the time when experiments were conducted.

For intra-specific signalling experiments, 12 pieces were cut from each seaweed specimen (6 *F. spiralis* specimens) and equally allocated to the upstream and downstream compartments of one control and one treatment aquarium, i.e. 3 pieces in each compartment (Fig. 3A), resulting in a total of 48 aquaria (2 treatments x 3 species x 8 replicates) at each site. In inter-specific signalling experiments, 12 pieces were cut from one specimen of (i) *S. muticum*, (ii) *H. siliquosa* or *C. humilis*, and (iii) *F. vesiculosus* or 6 *F. spiralis* individuals in the first, second and third sub-experiment, respectively. These pieces were equally allocated to the upstream compartment of four aquaria ( = donor pieces). In addition, 6 pieces were cut from each con- or hetero-familiar specimen and were equally allocated to the downstream compartment ( = recipient pieces) of 2 aquaria (Fig. 3B). This resulted in a total of 96 aquaria (2 grazing treatments x 2 recipient species x 3 donor species x 8 replicates) at each site. Although inter-specific signalling sub-experiments were conducted at slightly different times, each species performed comparably in the sub-experiments, as indicated by insignificant differences in growth rates of pieces of the same seaweed species between sub-experiments (Student’s t-test: all t_7_<1.931, p>0.05). Following their allocation to aquaria, the seaweed pieces remained there without grazers during the next 4 days (acclimation phase). This allowed seaweeds to acclimate to laboratory conditions and to reduce putative induced anti-herbivory defences acquired during their unknown grazing history in the field, as shown for *F. vesiculosus* and/or recover from changes in palatability that might have occurred in response to cutting [46]. Cutting, however, did not alter the palatability of *F. vesiculosus* [31] and other species [47] to isopods. Moreover, prior to their use in experiments all grazers were kept on a mixed algal diet (excluding seaweed species used in the induction phase) in a separate aerated 50 L container with seawater flow-through. On day 5, i.e. at the beginning of the induction phase, 4 isopods were added to the upstream compartment of a randomly selected half of aquaria of each treatment in both intra-specific and inter-specific signalling experiments. The remaining 8 aquaria of each treatment were kept without grazers ( = controls, Fig. 3). Selected grazer density matched naturally observed levels of isopod density on *F. vesiculosus* and *S. muticum* at Helgoland [48], as well as on *S. muticum* and *C. humilis* in Portugal (unpubl. data AE Engelen). Survival of isopods was recorded at least twice daily and, when appropriate, dead isopods were replaced by live con-specifics. After 12 days, the induction phase was terminated by removing isopods from the set-up and using seaweed pieces from downstream compartments in two-choice feeding assays. Previous studies demonstrated that 10 to 14 days of *I. baltica* grazing are needed to lower *F. vesiculosus* palatability [49], [50]. Extending the period of *I. baltica* grazing had been shown to cause fluctuations in *F. vesiculosus* palatability but did not increase the efficacy of anti-herbivory responses (unpubl. data CR Flöthe).

Two types of feeding assays tested whether the palatability of seaweed pieces positioned downstream of grazed con- or hetero-specifics ( = treated) was lower than that of pieces positioned downstream of non-grazed con- or hetero-specifics ( = control). The first type of feeding assay used fresh seaweed pieces, testing for the induction of morphological and/or chemical anti-herbivory defences. Thus, two seaweed pieces were taken from each downstream compartment of one treatment and control aquarium to which pieces originating from the same specimen, i.e. genetically identical pieces, had been allocated at the beginning of the experiments. The remaining third piece from each downstream compartment was stored at −80°C and used later in reconstituted food assays (see below). Subsequently, algal pieces were spun 10 times in a salad spinner, blotted with paper towels for 15 sec, and weighed separately on a balance (Sartorius LE323S, Germany) to the nearest 0.001 g before transferring one treated and one control piece to each of two feeding arenas ( = 200 mL glass Petri dish, experimental unit = EU). The assay started after adding 2 naïve isopods to one feeding arena. No grazers were added to the second feeding arena to assess non-feeding related ( = autogenic) changes in wet mass of fresh algae in the first feeding arena during the assay (Fig. 3). Seawater in feeding arenas was exchanged twice daily to reduce artefacts on grazer consumption, e.g. waste products accumulating in feeding arenas. At the end of 3 d feeding assays, each algal piece was reweighed following the above description. Isopod consumption of treated and control pieces was estimated as: B_start_ x (A_end_/A_start_) – B_end_, where B_start_ and B_end_ represent the initial and final wet mass of an assayed piece, respectively, and A_start_ and A_end_ represent the initial and final mass of the autogenic control piece, respectively [51]. A significantly higher consumption of control than of treated algal pieces was interpreted as an induction of anti-herbivore defences.

The second type of feeding assays used reconstituted food, testing for the induction of chemical anti-herbivory defences. After the induction phase, seaweed pieces that were stored at −80°C, were freeze-dried, ground to a homogenous fine powder, and 0.2 g of this powder were suspended in 1 ml of distilled water. This algal suspension was mixed with molten agar (0.043 g in 1.2 ml of distilled water) after the agar had cooled to 55°C, poured over a mosquito net (1 mm^2^ mesh size), and flattened between two glass plates [52]. After solidification, pellets of 15×15 mm^2^ were cut from algae-agar mixtures and marked with different incision patterns to distinguish between control and treated pellets. The control and treated pellet originating from the same seaweed specimen were transferred to one feeding arena (Fig. 3). Assays started after adding 2 naïve isopods and were terminated 36 h later. Prior to weighing, excessive water was removed from each pellet by blotting pellets with paper towels for 5 sec. Special care was taken to ensure that no pellet material was removed during blotting. Set-up and all other conditions were identical to assays using fresh seaweed pieces. Isopod consumption was calculated as the difference in wet mass of a food pellet between start and end of a feeding assay.

### Statistical Analysis

Differences in isopod consumption between treated and control pieces were analysed with paired t-tests after confirming normal distribution of differences between the consumption of treated and control pieces with a Kolmogornov-Smirnov test. Despite the large number of paired t-tests (i.e. 18), sequential Bonferroni adjustments were not carried out because χ^2^ tests indicated that the number of observed significant paired t-tests in assays with fresh (i.e. 5) or reconstituted food (i.e. 6) was significantly different from what could be expected by chance (i.e. 18×0.05 = 0.9; fresh food: χ^2^ = 16.94, p<0.0001, reconstituted food: χ^2^ = 26.47 p<0.0001).
